# Antibacterial use in Norwegian horses: a nationwide registry-based cross-sectional study

**DOI:** 10.1186/s12917-026-05474-z

**Published:** 2026-04-16

**Authors:** Leif Lukas Löfling, Caroline S. Haadem, Arvid Reiersen, Stefan Börjesson, Kari Olli Helgesen

**Affiliations:** 1 Section for Epidemiology, Department of Health Data, Norwegian Veterinary Institute, Postboks 64, Ås, 1431 Norway; 2 Equine Clinic Section, Department of Companion Animal Clinical Sciences, Faculty of Veterinary Medicine, Norwegian University of Life Sciences, Ås, Norway; 3 Section for Terrestrial Animal Health and Welfare, Department of Animal Health, Norwegian Veterinary Institute, Ås, Norway; 4 Section for Veterinary Public Health, Department of Animal Health, Norwegian Veterinary Institute, Ås, Norway

**Keywords:** Antibacterial use, Horse, Population-based, Registry, Drug utilization, Data quality, Data accuracy, Data completeness

## Abstract

**Background:**

Antibacterials are essential for treating infections in horses, but the use contributes to antimicrobial resistance (AMR), which threatens animal and human health. Despite these concerns, antibacterial use in horses has not been systematically studied and described in Norway, and nationwide registry-based analyses in other countries are scarce. The aim of this study is to describe antibacterial use in Norwegian horses, 2022–2024, by analysing nationwide registry data, with a particular emphasis on gynecological and obstetrical conditions. In addition, we will evaluate the data quality in light of the upcoming EU reporting requirements for data on antibacterial use in horses.

**Results:**

A total of 30,362 records reported to the Norwegian Veterinary Prescription Registry (VetReg) were included. Of these, 42.5% were reported by veterinarians and 57.5% by pharmacies. The two most common diagnostic groups treated were skin and hoof conditions and musculoskeletal conditions, accounting for approximately half of all records. Gynecological and obstetrical conditions accounted for 4% of the records.

Antibacterial use (in kg active substance) was dominated by oral paste containing the combination of sulphonamide and trimethoprim (> 90% in all three years), followed by injections with beta-lactamase sensitive penicillins. AMEG category D antibacterials (prudent use) accounted for > 99% of the use. These patterns were also observed for gynecological and obstetrical conditions.

The completeness of data on antibacterial use in horses in VetReg, in relation to sales data, increased from 75.5% in 2022 to 84.7% in 2024.

**Conclusion:**

A higher proportion of AMEG category D antibacterials (> 99%) was observed in our study than in Spain (85%). However, increasing carriage of resistant *E. coli* in the general horse population suggests that the extensive use of sulphonamide + trimethoprim should be further evaluated. Prudent use could be evaluated to some extent, but the reporting of general organ-related diagnostic codes limited this evaluation. Lack of fully reliable population data for horses limits how differences in antibiotic use between years and countries can be described and evaluated. VetReg data quality for horses was in line with or better than for the species for which data are already reported to the European Medicines Agency.

**Supplementary Information:**

The online version contains supplementary material available at 10.1186/s12917-026-05474-z.

## Background

Antibacterials are essential for treating infections in both humans and animals. However, use of antibacterials will select for antimicrobial resistance (AMR) and may compromise the effectiveness of antibacterials [1]. It is essential to have effective treatment against infections for horses and continued rise in resistance may limit the treatment options. Thus, leading to increased morbidity and mortality, and negatively impact horse welfare and the economy of horse enterprises. In addition, close contact between horses and humans may also facilitate transmission of AMR bacteria from horses to humans, and vice versa [2]. Specific equine facilities where antibacterials are used and with a high flow of horses, such as horse clinics and stud farms, are at particular risk to become contaminated with and thus facilitate dissemination of AMR bacteria. Despite its importance, antibacterial use in horses has not yet been systematically studied and described in Norway. Furthermore, the use of nationwide registry data to study antibacterial use in horses remains scarce in the available literature. We have only been able to identify two such studies, from Spain and Estonia, presented as annual reports and as an interactive dashboard [3–5], although not available in English or as peer-reviewed scientific articles. Previous peer-reviewed studies on antibacterial use in horses have relied on non-nationwide data sources, such as clinical records collected from a single clinic or limited number of clinics [6–8]. Utilization studies are important for assessing the prudence of current practices and identifying areas where regulatory or management interventions may be warranted. Hence, studies based on nationwide data are needed for evaluations of use and for providing knowledge for interventions at a national level. In contrast, studies relying on more limited datasets often raise concerns about generalizability when their findings are applied in a broader context. The hindsight of using nationwide registry data compared to clinical records might be that registry data have limited details of e.g. diagnosis, and that it is difficult to separate use in more high AMR risk equine facilities, such as horse clinics and stud farms, from general field practice use.

Within horse breeding, endometritis (a superficial inflammation involving the endometrium) is a common condition occurring in relation to breeding and is a common cause of reduced fertility in the mare [9–11]. Acute metritis (a severe gynecological infection involving all layers of the uterus, i.e., endometrium, myometrium and perimetrium) post-partum can be life-threatening for the mare. Metritis should always be treated aggressively, including using antibacterials, while antibacterial treatment of endometritis is not always needed and might even be contraindicated [12]. Knowledge and subsequent evaluation of the treatment of these conditions are therefore of importance with regards to horse health and fertility. It is also important from an AMR perspective because breeding mares are more likely to visit stud farms than other horses. The bacteria most frequently causing endometritis and metritis are Enterobacterales (e.g., *Escherichia coli*) and Bacillales (e.g., *Staphylococci* or *Streptococci*). Some Enterobacterales have developed resistance to third and fourth generation cephalosporins and/or carbapenems, so called extended spectrum beta-lactamase producing (ESBL) bacteria. These bacteria are also often multidrug-resistant (MDR), i.e. resistant to three or more antibacterial classes [13]. Third and fourth generation cephalosporins are considered among the highest priority critically important antimicrobials in human medicine according to the World Health Organization. For veterinary medicine, the recommendation is to restrict the use of these antibacterials in accordance with the Antimicrobial Advice ad hoc Expert Group (AMEG) Categorisation published by the European Medicines Agency (EMA) [14, 15]. Carbapenems are banned for use in animals in Europe [16]. Another concerning bacterium in humans and horses is methicillin-resistant *Staphylococcus aureus* (MRSA) [17], which is resistant to all clinically available beta-lactam antibacterials (e.g., penicillin, cephalosporins, carbapenems), and often MDR [18]. Although so far ESBL producing *E. coli* and MRSA appears rare in the Norwegian horse population [19].

For assessment of prudent use and adherence to treatment guidelines, access to information on specific diagnoses is important. Grave et al., 2019 [20], found almost exclusively general organ-related diagnoses in their study of antibacterial use in dogs in Norway, but they included only records reported by pharmacies on dispensing to animal owners in the Veterinary prescription registry (VetReg) and not records on use reported by veterinarians. For prescriptions, the national competent authority asks for these general organ-related diagnosis [21]. Veterinarians will use more specific diagnosis in their Practice Management Software (PMS) and hence data of veterinary use (as opposed to prescriptions) might include more precise diagnosis and, therefore be better for evaluating prudent use. Data on veterinarians’ use are believed to be a larger proportion of use data for horses compared to dogs, because it is mandatory for veterinarians to register these data [22], as opposed to for dogs where it is currently still voluntary. From 2029 onwards collecting use data for dogs becomes mandatory for all European Union (EU) and European Economic Area (EEA) countries according to EU regulation 2019/6 on veterinary medicinal products [23]. This regulation also makes it mandatory for EU and EEA countries to collect annual antibacterial use data for horses from 2026 to be reported to EMA.

The aim is to collect harmonized data across Europe to monitor and reduce AMR, enable comparable analysis across countries and support evidence-based policy making. Data to be reported should be “accurate, complete and consistent” [24]. In Norway it has been mandatory to report medicinal use data for horses to VetReg since 2012 [22]. The horse data in VetReg have not been evaluated in terms of compliance with the EU regulations. Quality evaluations of antibacterial use data in VetReg have however been performed for the species for which data are currently reported to EMA (i.e. cattle, pigs, chicken and turkeys), horses (only completeness assessment of antibacterial use data for 2016), and vaccine data for fish [12, 25, 26]. In these evaluations several quality issues related to completeness and data accuracy have been identified.

The aim of this study is to analyse nationwide registry data to describe antibacterial use in horses in Norway, with a particular emphasis on category A (avoid) and B (restrict) antibacterials according to EMA’s AMEG categorisation. Use for gynecological and obstetrical conditions will further be described more thoroughly, as an example of a group of diagnosis, to evaluate to what extent data from VetReg can be used to assess if the antibacterial use followed the national treatment guidelines. In addition, the aim is to evaluate if the national data registry was in adherence with the requirements in upcoming EU regulation on data reporting of antibacterial use in horses.

## Methods

### Data sources

**Fig. 1:**
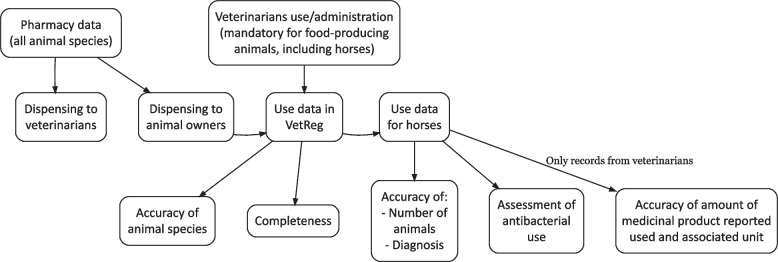
Illustration of data used in the different analyses of the study

This nationwide registry-based drug utilization study included data on antibacterial use in horses in Norway between 2022 and 2024, obtained from VetReg. VetReg is owned by the Norwegian Food Safety Authority and made available for the Norwegian Veterinary Institute through an agreement [22]. For this study, the data were downloaded from the Norwegian Food Safety Authority on 18.03.2025. Since 2012, pharmacies and veterinarians have been legally required to report information on medicines to VetReg [22]. Pharmacies must report all medicines (both veterinary and human medicinal products) dispensed to animal owners (i.e., all animal species) and to veterinarians for use in clinical practice (Fig. 1). Veterinarians must report all use and administration of medicines in food-producing animals, including horses, while reporting for use in other animals kept or bred (e.g., companion animals and fur animals) remains currently still voluntary.

In VetReg, one record contains information about the dispensing or use of one medicinal product on one occasion to one animal or a group of animals, which applies to both records from pharmacies and veterinarians. Medications are specified in VetReg by product name, Anatomical Therapeutic Chemical (ATC) code/ATC codes for veterinary medicinal products (ATCvet) and Nordic article number (unique identifier for a specific package size of a medicinal product). In addition, VetReg includes information on, for example, amount and unit of amount of the medicinal product dispensed or used, number of packages dispensed from pharmacies, reporting source (pharmacy or veterinarian), date of dispensing or use, date of reporting to VetReg, animal species/category, number of animals treated and diagnosis. Full list of variables in VetReg is available in Udhwani et al., 2025 [26]. The diagnostic code assigned in VetReg can be a general organ-related code or a more specific code (i.e., a single diagnosis or a group of closely related diagnoses) [21, 27]. Sales data for veterinary medicinal products in Norway during the study period (2022–2024) were obtained from the Norwegian Institute of Public Health. Wholesalers are legally required to report their sales of medicinal products to pharmacies directly to the Norwegian Institute of Public Health [28]. Sales data includes the number of sold packages per Nordic article number and were used when estimating the completeness of VetReg.

To supplement VetReg and sales data, information on active substance, strength of active substance, pharmaceutical form and package size were obtained from the publicly available FEST-database (in Norwegian: Forskrivnings-og ekspedisjonsstøtte, abbreviated FEST). The FEST-database is owned by the Norwegian Medical Products Agency and is primarily set up to support the prescribing and dispensing of medicines in Norway.

### Assessment of antibacterial use

The antibacterials assessed in this study were all antibacterials (i.e., veterinary and human medicinal products) for systemic, intramammary and intrauterine use. The ATC and ATCvet codes used to identify the antibacterials in VetReg are listed in Supplementary Table 1. These codes were identical to those for which data is mandatory to report to EMA from the reporting year 2026 plus the codes QJ04, J04 and QJ54 [24].

Antibacterial use was assessed separately for each year and stratified by pharmaceutical form, antibacterial class and AMEG category. These categories are A: avoid use, B: restrict use, C: use with caution, D: prudent use [14]. For each stratification, two indicators were used: (1) percentage of the total amount of active substance, and (2) percentage of the total number of records reported to VetReg. This was performed in the complete dataset and separately for the records in which gynecological and obstetrical conditions were the indication for treatment. In general, the amount of active substance used per record was calculated by multiplying the reported amount of medicinal product used with the strength of the active substance(s) [25].

We also assessed which diagnoses were given for treatment with antibacterials in horses, reporting the percentage of records per diagnosis or diagnostic group. This was conducted across diagnostic groups, diagnostic groups treated with antibacterials of AMEG A or B category, and specific gynecological and obstetrical conditions. Diagnoses were grouped into diagnostic groups in accordance with Supplementary Table 2.

### Quality evaluation

Because VetReg data has not been previously used for assessing antibacterial use in horses and because of the new EU-regulation (2019/6) on reporting use data for horses [23], and its underlying regulation (2021/578) specifying that the reported data should be “accurate, complete and consistent” [24], a quality evaluation of the data was performed. To evaluate the quality of the data on antibacterial use in horses reported to VetReg, two aspects of quality were evaluated: accuracy and completeness.

Accuracy refers to whether the variables reported to VetReg represents the true value. Records for use data (i.e., reported dispensing by pharmacies to animal owners and reported use/administration by veterinarians) were included when evaluating accuracy. The following variables were evaluated for accuracy: (1) animal species (the proportion of records missing information on species); (2) number of animals (the proportion of records missing information on number of animals); (3) amount of medicinal product reported used and the associated unit per pharmaceutical form (the proportion of records where the amount used could be calculated without any correction of the unit, records calculated after changing the unit, and records presumed inaccurate and excluded) and (4) diagnosis (the proportion of records missing diagnostic information and the proportion of records with a diagnosis not relevant for horses).

All records on use (i.e., for all animal species) were included in the evaluation of accuracy of the animal species variable (Fig. 1). While, when evaluating accuracy of the other variables (i.e., number of animals, reported amount of medicinal product used and the associated unit reported, and diagnosis), only records on use in horses were included.

For the evaluation of accuracy of the reported amount of medicinal product used and the associated unit reported, only records reported by veterinarians were included. Pharmacy records were not included as pharmacies report their dispensing almost exclusively in number of packages and the reporting is linked to the pharmacy management system. Given that oral paste and injections are the predominant pharmaceutical forms in veterinarian-reported records, the evaluation focused on these two pharmaceutical forms. As part of assessing accuracy of the reported amount, data cleaning was performed at record level to correct presumed inaccuracies in the reported unit. To calculate the amount of medicinal product used per animal per record, the total amount reported per record was divided by the reported number of animals indicated to be treated. For records with missing information on number of animals, we assumed that one horse was treated. To identify records with reported use considered to be unreasonably high and therefore inaccurate, we applied a set of predefined criteria. These criteria varied by species (species other than horses were relevant for the assessment of completeness) and pharmaceutical forms. For horses, Grubbs’ test was used to identify outliers (p-value < 0.01) for each combination of Nordic article number and reporting unit [29]. Records identified as outliers, as well as records for combinations of Nordic article number and reporting unit with less than seven records (Grubbs’ test cannot be performed with less than seven observations), underwent manual review. In the manual review, records were excluded if the reported amount per animal exceeded a cut-off value. This cut-off was calculated as twice the highest dose specified in the summary of product characteristics multiplied by the maximum treatment duration for a horse with the highest assumed weight (1,300 kg). When horse-specific dosing information was unavailable in the summary of product characteristics, cattle dosing was used as a proxy. This choice was made to simplify the methodology and since the veterinarians’ rationale for dosing when the product was not marketed for horses was unknown and could vary. Details on the methodology applied to other species relevant for completeness assessment are available in Udhwani et al., 2025 [26].

Completeness was assessed by evaluating the extent to which data on use of antibacterial veterinary medicinal products for horses was entered into VetReg. This was achieved by comparing VetReg data with national sales data. Detailed methodology is described in Udhwani et al., 2025 [26]. The completeness evaluation included antibacterial veterinary medicinal products for which reported use in horses accounted for > 85% of the total reported use in the specific year (Fig. 1). To assess the completeness of use data, reported use of these veterinary medicinal products for any species (dispensed by pharmacies to animal owners and used/administrated by veterinarians) was compared to the sales data. For evaluating the completeness of pharmacy data, the antibacterials dispensed by pharmacies (i.e., to veterinarians and animal owners) was compared to the sales data.

All data management and analysis were performed using R Statistical software version 4.4.2 [30].

## Results

### Overall use

A total of 30,480 antibacterial records for horses were reported to VetReg between 2022 and 2024. Of these, 118 veterinary records were excluded; 94 because the reported amount was considered to be too high after manual review, and 24 because amount of active substance could not be calculated. The final dataset comprised 30,362 records (2022: 10,416; 2023: 10,369; 2024: 9,577), of which 17,467 (57.5%) were records of dispensing by pharmacies to animal owners and 12,895 (42.5%) were records of use or administration by veterinarians. The total amount of active substance used was 973 kg in 2022, 997 kg in 2023 and 975 kg in 2024.

**Fig. 2:**
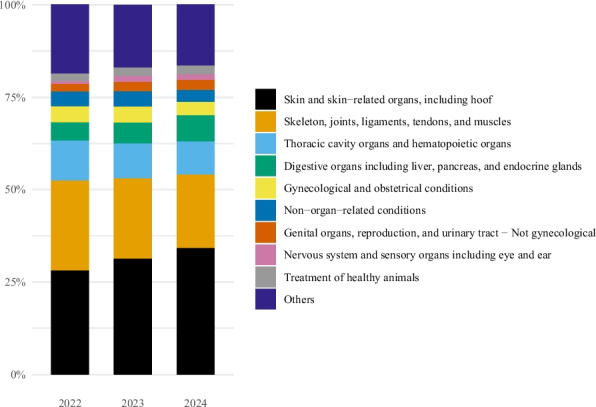
Diagnostic groups assigned for treatment with antibacterials in horses in Norway by year, 2022–2024. Percentage of records assigned the diagnostic group. Data comes from the Veterinary Prescription Registry. Skin and skin − related organs, including hoof (2022: 28.1%, 2023: 31.2%, 2024: 34.1%). Skeleton, joints, ligaments, tendons, and muscles (2022: 24.4%, 2023: 21.9%, 2024: 20.0%). Thoracic cavity organs and hematopoietic organs (2022: 10.7%, 2023: 9.3%, 2024: 8.9%). Digestive organs including liver, pancreas, and endocrine glands (2022: 5.1%, 2023: 5.8%, 2024: 7.2%). Gynecological and obstetrical conditions (2022: 4.3%, 2023: 4.3%, 2024: 3.6%). Non − organ − related conditions (2022: 3.9%, 2023: 4.1%, 2024: 3.1%). Genital organs, reproduction, and urinary tract − Not gynecological (2022: 2.2%, 2023: 2.6%, 2024: 2.8%). Nervous system and sensory organs including eye and ear (2022: 0.7%, 2023: 1.6%, 2024: 1.6%). Treatment of healthy animals (2022: 2.0%, 2023: 2.2%, 2024: 2.2%). Others (2022: 18.7%, 2023: 17.0%, 2024: 16.6%)

In all three years, the two most common diagnostic groups indicated for antibacterial use were conditions affecting the skin and related structures (including hooves) and the musculoskeletal system—with 28.1% and 24.4% of the records in 2022, 31.2% and 21.9% in 2023, and 34.1% and 20.0% in 2024, respectively (Fig. 2). For skin-related conditions, the proportion of records with a general organ-related diagnostic code decreased from 77.5% in 2022 to 72.3% in 2023 and 68.4% in 2024. For musculoskeletal conditions, these proportions were 65.8%, 78.3% and 69.2%, respectively.

**Fig. 3:**
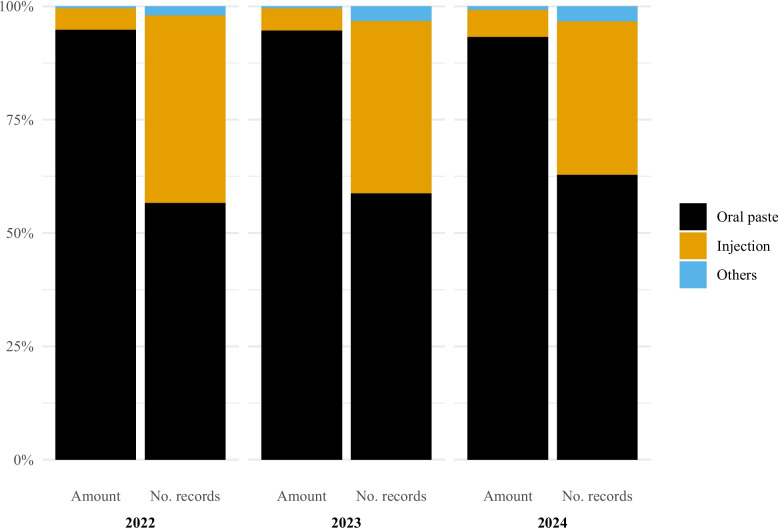
Antibacterial use in horses in Norway by pharmaceutical form and year, 2022–2024. Left bar is percentage of amount of active substance and right bar is percentage of records. Data comes from the Veterinary Prescription Registry. Oral paste (Amount of active substance: 2022: 94.8%, 2023: 94.6%, 2024: 93.2%; Number of records: 2022: 56.6%, 2023: 58.7%, 2024: 62.8%). Injection (Amount of active substance: 2022: 4.8%, 2023: 5.0%, 2024: 6.0%; Number of records: 2022: 41.4%, 2023: 38.1%, 2024: 33.9%). Others (Amount of active substance: 2022: 0.4%, 2023: 0.4%, 2024: 0.8%; Number of records: 2022: 2.0%, 2023: 3.2%, 2024: 3.3%)

Antibacterial use in horses was almost exclusively oral paste containing a sulphonamide (sulfadiazine) and trimethoprim (all oral paste used in horses in the study period contained this combination). This combination in the form of oral pastes accounted for 94.8% of the amount of active substance in 2022 (56.6% of records), 94.6% (58.7% of records) in 2023 and 93.2% (62.8% of records) in 2024 (Fig. 3).

**Fig. 4:**
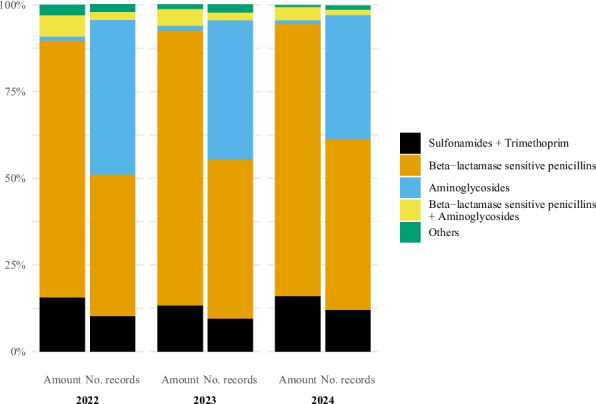
Antibacterial injection use in horses in Norway by antibacterial class and year, 2022–2024. Left bar is percentage of amount of active substance and right bar is percentage of records. Data comes from the Veterinary Prescription Registry. Sulfonamides + Trimethoprim (Amount of active substance: 2022: 15.5%, 2023: 13.2%, 2024: 15.9%; Number of records: 2022: 10.1%, 2023: 9.5%, 2024: 12.0%). Beta − lactamase sensitive penicillins (Amount of active substance: 2022: 74.1%, 2023: 79.3%, 2024: 78.6%; Number of records: 2022: 40.9%, 2023: 45.8%, 2024: 49.2%). Aminoglycosides (Amount of active substance: 2022: 1.3%, 2023: 1.6%, 2024: 1.1%; Number of records: 2022: 44.7%, 2023: 40.3%, 2024: 36.0%). Beta − lactamase sensitive penicillins + Aminoglycosides (Amount of active substance: 2022: 6.2%, 2023: 4.8%, 2024: 3.9%; Number of records: 2022: 2.3%, 2023: 2.2%, 2024: 1.5%). Others (Amount of active substance: 2022: 2.9%, 2023: 1.2%, 2024: 0.4%; Number of records: 2022: 2.2%, 2023: 2.3%, 2024: 1.2%)

Injection was the second most common pharmaceutical form (Fig. 3). In terms of amount of active substance, the two most used antibacterial classes for injections were beta-lactamase sensitive penicillins (2022: 74.1%, 2023: 79.3%, 2024: 78.6%) and sulphonamide + trimethoprim (2022: 15.5%, 2023: 13.2%, 2024: 15.9%) (Fig. 4). When considering the number of records, the two most common classes were aminoglycosides (2022: 44.7%, 2023: 40.3%, 2024: 36.0%) and beta-lactamase sensitive penicillins (2022: 40.9%, 2023: 45.8%, 2024: 49.2%).

**Fig. 5:**
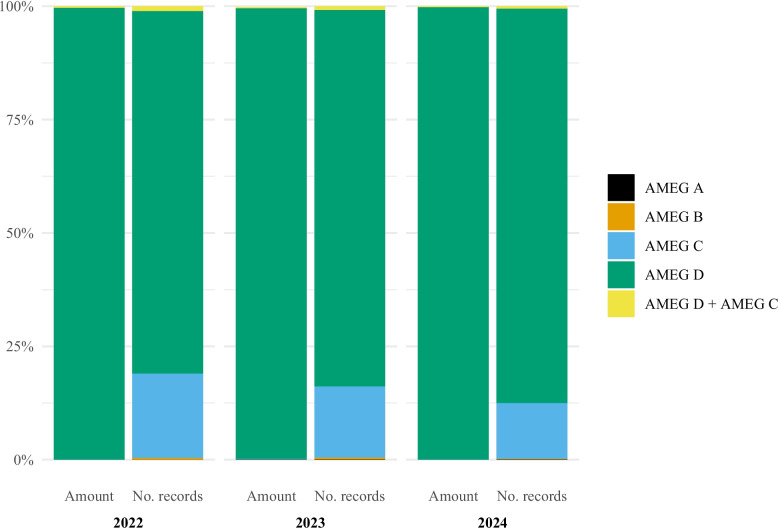
Antibacterial use in horses in Norway by AMEG category and year, 2022–2024. Left bar is percentage of amount of active substance and right bar is percentage of records. Data comes from the Veterinary Prescription Registry. AMEG (Antimicrobial Advice Ad Hoc Expert Group). AMEG A (Amount of active substance: 2022: < 0.1%, 2023: 0.1%, 2024: < 0.1%; Number of records: 2022: < 0.1%, 2023: 0.1%, 2024: 0.1%). AMEG B (Amount of active substance: 2022: < 0.1%, 2023: < 0.1%, 2024: < 0.1%; Number of records: 2022: 0.4%, 2023: 0.4%, 2024: 0.1%). AMEG C (Amount of active substance: 2022: 0.1%, 2023: 0.1%, 2024: 0.1%; Number of records: 2022: 18.6%, 2023: 15.7%, 2024: 12.3%). AMEG C + D (Amount of active substance: 2022: 0.3%, 2023: 0.2%, 2024: 0.2%; Number of records: 2022: 1.0%, 2023: 0.8%, 2024: 0.5%). AMEG D (Amount of active substance: 2022: 99.6%, 2023: 99.4%, 2024: 99.7%; Number of records: 2022: 80.0%, 2023: 83.0%, 2024: 87.0%)

Almost all antibacterials used were classified as AMEG category D (prudent use), accounting for more than 99% of the amount of active substance each year (Fig. 5). When considering the number of records, AMEG category D represented 80.0% in 2022, 83.0% in 2023, and 87.0% in 2024.

AMEG category A (avoid use) antibacterials were reported in 19 records during the study period. Rifampicin was the only AMEG category A antibacterial used. Of these, ten (52.6%) were indicated for treating conditions affecting the thoracic cavity and hematopoietic organs, one (5.3%) for digestive organs including liver, pancreas, and endocrine glands, and eight (42.1%) for other unspecified conditions. None of these records were assigned a disease specific code.

Antibacterials categorised as AMEG B (restrict use) were reported used in 0.4% of the records in 2022 and 2023, and 0.1% in 2024 (Fig. 5). Enrofloxacin was the only AMEG category B antibacterial used. The most common diagnostic groups indicated for use of category B antibacterials were musculoskeletal conditions (28.4%) and conditions affecting the thoracic cavity and hematopoietic organs (25.9%) (Supplementary Fig. 1). The most common more specific disease codes assigned for treatment with AMEG category B were respiratory infections (n = 13) and colic/abomasum displacement (n = 9).

Treatment of healthy animals were reported as the indication in 2.0% of the records in 2022 and 2.2% in both 2023 and 2024 (Fig. 2). For treatment of healthy horses, oral paste (sulphonamide [sulfadiazine] + trimethoprim) and injections were the most common pharmaceutical forms. Oral paste containing sulphonamide + trimethoprim accounted for more than 90% of the amount of active substance used for treatment of healthy animals in all three years, and represented 44.9% of the records in 2022, 47.1% in 2023 and 48.4% in 2024. Injections accounted for 9.2% of the amount of active substance in 2022 (49.8% of records), 9.0% (45.7% of records) in 2023 and 5.4% (44.6% of records) in 2024.

### Gynecological and obstetrical conditions

**Fig. 6:**
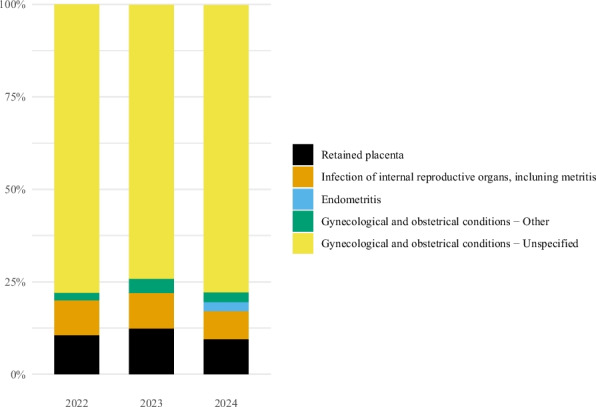
Diagnoses assigned for antibacterial treatment of gynecological and obstetrical conditions in horses in Norway, 2022–2024. Percentage of records assigned the diagnosis. Data comes from the Veterinary Prescription Registry. Retained placenta (2022: 10.5%, 2023: 12.3%, 2024: 9.4%). Infection of internal reproductive organs, including metritis (2022: 9.4%, 2023: 9.6%, 2024: 7.6%). Endometritis (2022: 0.0%, 2023: 0.0%, 2024: 2.6%). Gynecological and obstetrical conditions − Other (2022: 2.2%, 2023: 4.0%, 2024: 2.6%). Gynecological and obstetrical conditions – Unspecified (2022: 77.9%, 2023: 74.0%, 2024: 77.6%)

Gynecological and obstetrical conditions were the indication in 4.3% of the records in both 2022 and 2023, and 3.6% in 2024 (Fig. 2). In all three years, more than 70% of the gynecological and obstetrical conditions were unspecified (Fig. 6). The two most commonly specified conditions were retained placenta and infections of internal reproductive organs (including metritis), representing 10.5% and 9.4% of the gynecological and obstetrical records in 2022, 12.3% and 9.6% in 2023, and 9.4% and 7.6% in 2024. Endometritis was not reported as indication for antibacterial treatment in horses in 2022 or 2023; however, in 2024 it was reported in nine (2.6%) of the records for gynecological and obstetrical conditions.

**Fig. 7:**
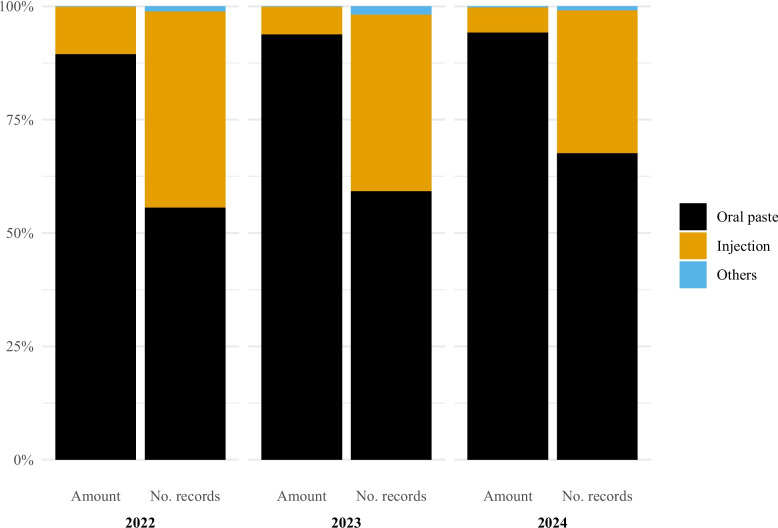
Antibacterial use for treatment of gynecological and obstetrical conditions in horses in Norway by pharmaceutical form and year, 2022–2024. Left bar is percentage of amount of active substance and right bar is percentage of records. Data comes from the Veterinary Prescription Registry. Oral paste (Amount of active substance: 2022: 89.4%, 2023: 93.8%, 2024: 94.2%; Number of records: 2022: 55.6%, 2023: 59.2%, 2024: 67.6%). Injection (Amount of active substance: 2022: 10.5%, 2023: 6.1%, 2024: 5.6%; Number of records: 2022: 43.3%, 2023: 39.0%, 2024: 31.5%). Others (Amount of active substance: 2022: 0.1%, 2023: 0.1%, 2024: 0.2%; Number of records: 2022: 1.1%, 2023: 1.8%, 2024: 0.9%)

**Fig. 8:**
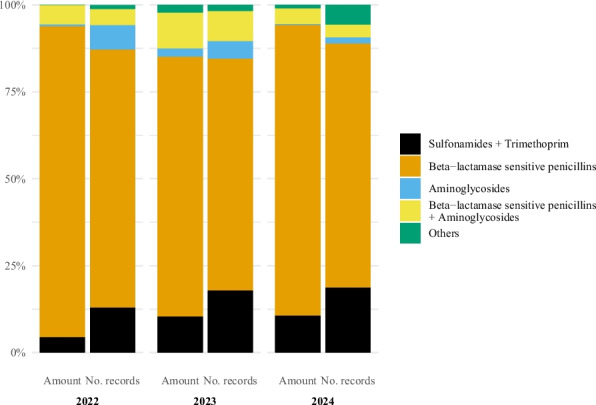
Antibacterial injection use for treatment of gynecological and obstetrical conditions in horses in Norway by antibacterial class and year, 2022–2024. Left bar is percentage of amount of active substance and right bar is percentage of records. Data comes from the Veterinary Prescription Registry. Sulfonamides + Trimethoprim (Amount of active substance: 2022: 4.4%, 2023: 10.3%, 2024: 10.6%; Number of records: 2022: 12.9%, 2023: 17.8%, 2024: 18.7%). Beta − lactamase sensitive penicillins (Amount of active substance: 2022: 89.4%, 2023: 74.8%, 2024: 83.6%; Number of records: 2022: 74.2%, 2023: 66.7%, 2024: 70.1%). Aminoglycosides (Amount of active substance: 2022: 0.7%, 2023: 2.5%, 2024: 0.4%; Number of records: 2022: 7.2%, 2023: 5.2%, 2024: 1.9%). Beta − lactamase sensitive penicillins + Aminoglycosides (Amount of active substance: 2022: 5.5%, 2023: 10.2%, 2024: 4.5%; Number of records: 2022: 4.6%, 2023: 8.6%, 2024: 3.7%). Others (Amount of active substance: 2022: < 0.1%, 2023: 2.2%, 2024: 0.9%; Number of records: 2022: 1.0%, 2023: 1.7%, 2024: 5.6%)

For antibacterial treatment of gynecological and obstetrical conditions in horses, oral paste containing sulphonamide (sulfadiazine) and trimethoprim accounted for 89.4% of the amount of active substance in 2022 (55.6% of records), 93.8% (59.2% of records) in 2023, and 94.2% (67.6% of records) in 2024 (Fig. 7). Injection was the second most frequently used pharmaceutical form. Among injections, beta-lactamase sensitive penicillins and sulphonamide in combination with trimethoprim were the predominant antibacterial classes. These classes accounted for 89.4% and 4.4% of the amount of active substance in 2022, 74.8% and 10.3% in 2023, and 83.6% and 10.6% in 2024 (Fig. 8). When considering the number of records, they represented 74.2% and 12.9% of the records in 2022, 66.7% and 17.8% in 2023, and 70.1% and 18.7% in 2024.

For treatment of endometritis, oral paste (sulphonamide + trimethoprim) was reported used in five (55.6%) of the nine endometritis records, accounting for 97.9% of the amount of active substance used. In addition to oral paste, endometritis was treated with injections containing ampicillin or benzylpenicillin.

### Quality evaluation

#### Accuracy

Information on animal species was missing for 0.1% of the records in 2022 and 2023, and for < 0.1% in 2024. All other variables evaluated for accuracy constituted of horse records only. The proportion of records with missing information on the number of animals was 42.4% in 2022 (veterinarians: 91.2%; pharmacies: < 0.1%), 41.1% in 2023 (veterinarians: 93.2%; pharmacies: 1.9%) and 54.2% in 2024 (veterinarians: 94.7%; pharmacies: 28.9%).

For injections, the amount used could be calculated without correction of the unit for 100% of the records in 2022, 97.9% in 2023 and 99.9% in 2024 (Supplementary Table 3). For oral paste, the corresponding proportions were 99.1% in 2022, 98.2% in 2023 and 98.2% in 2024.

Information on diagnosis was not missing for any records in 2022 and for < 0.1% of the records in 2023 and 2024 (Supplementary Table 4). The diagnosis given was not considered relevant for horses in 0.5% of the records in 2022, 0.6% in 2023 and 0.8% in 2024. A more specific code was given in 23.0% of the records in 2022 (veterinarians: 49.4%; pharmacies: 0.1%), 24.5% in 2023 (veterinarians: 50.8%; pharmacies: 4.8%) and 28.0% in 2024 (veterinarians: 61.0%; pharmacies: 7.4%). The rest of the diagnostic codes assigned for antibacterial treatment in horses in VetReg were general organ-related codes.

#### Completeness

The completeness evaluation included antibacterial veterinary medicinal products for which reported use in horses accounted for > 85% of the total reported use to VetReg. These products accounted for 95.1% of the amount of active substance used in horses in 2022 (nine products), 94.7% in 2023 (six products) and 94.0% in 2024 (six products). The overall completeness of use data (i.e., dispensed by pharmacies to animal owners and used/administrated by veterinarians) in terms of amount of active substance reported to VetReg was estimated to be 75.5% in 2022, 83.3% in 2023 and 84.7% in 2024 (Supplementary Table 5). The overall completeness of pharmacy data (i.e., dispensed by pharmacies to animal owners and veterinarians) reported to VetReg was estimated to be 75.6% in 2022, 83.5% in 2023 and 95.6% in 2024 (Supplementary Table 6).

## Discussion

Overall, the total amount of antibacterials (in kg active substance) remained stable throughout the study period, ranging from 973 to 997 kg. In all years, skin-related and musculoskeletal conditions were the two most common diagnostic groups indicated for treatment with antibacterials, together representing > 50% of the records. Oral paste containing sulfadiazine (a sulphonamide) + trimethoprim and injections containing beta-lactamase sensitive penicillins or sulphonamide + trimethoprim were consistently the predominant pharmaceutical forms and classes used in horses. This pattern was also observed for treating gynecological and obstetrical conditions. The only AMEG category A antibacterial used was rifampicin and enrofloxacin was the only AMEG category B antibacterial used. Rifampicin is classified as “critically important antimicrobials” for human medicine by the World Health Organization and enrofloxacin is classified as “highest priority critically important antimicrobials” for human medicine [15]. Approximately 2% of the records in VetReg were indicated for treatment of healthy animals. This could be antibacterial use for metaphylactic or preventive purpose or it may be incorrect registration of the diagnosis.

The Norwegian treatment guidelines presents a simplified summary of antibacterial use in horses in 2016, using VetReg data [12]. This summary reported that sulphonamides (with or without trimethoprim) accounted for 76% of the amount of active substance used in horses, followed by trimethoprim (15%), and beta-lactamase sensitive penicillins (7%). Our findings that sulphonamides + trimethoprim were the most common antibacterials used in horses also align with observations from previous peer-reviewed studies from other countries. However, in our study we observed a higher use of sulphonamides + trimethoprim (approximately 94% of active substance and 59% of treatment records) than reported in those studies. Schnepf et al. (2000), analysed data from 837 horses treated with antimicrobials (administrated or prescribed) in 2017 at the horse clinic at the Hannover University of Veterinary Medicine [6]. They reported that oral sulphonamides (with or without trimethoprim) were the most used antibacterial treatment in horses with 52% of the amount of active substance, followed by beta-lactamase sensitive penicillin given as injection (18%). Similar patterns were found in a larger German study by Merle et al. (2025) and in a study from the United Kingdom by Tallon et al. (2023) [7, 8]. Merle et al. used data from 57 veterinary practices from 2018 to 2023 [8]. Among 19,680 consultations resulting in antibacterial treatment (administrated or prescribed), sulphonamides + trimethoprim were most used (34% of treatment occasions), followed by aminopenicillins (30%) and aminoglycosides (18%). However, Merle et al. did not stratify antibacterial classes by pharmaceutical form. Tallon et al. assessed antibacterial treatment in horses in the United Kingdom from 2012 to 2021, using data from 13 practices in England and one in Wales, covering approximately 15% of the national horse population [7]. Sulphonamides were consistently the most used antibacterial throughout the study period, with 74% of the amount of active substance used (mg/kg biomass) in 2021, followed by tetracyclines (14%) and penicillin (7%) (no stratification of penicillins). When expressed as defined daily dose (DDDvet) per animal per year, sulphonamides were the most used in the early years followed by tetracyclines, but tetracyclines became the most used by the end of the study period (2021: 43%), followed by sulphonamides (2021: 37%).

In contrast to our findings, national reports from Estonia (2024 data) and Spain (2023 data) report that tetracyclines were the most used antibacterials in horses [3–5]. Furthermore, we observed a higher proportion of AMEG category D (prudent use) (> 99%) than Spain (85%). In Estonia, doxycycline (a tetracycline) was the most used antibacterial agent in horses with 36% of the amount of active substance used, followed by sulfadiazine (26%), other sulphonamides (19%), trimethoprim (9%), and benzylpenicillin (5%) [5]. The Estonian report did not provide data by pharmaceutical form. In Spain, tetracyclines (predominantly oxytetracycline) were the most used antibacterials in horses with 40% of the amount of active substance used in horses (mg/biomass), followed by benzylpenicillin (26%), sulphonamides (19%; predominantly sulfadiazine and sulfadimidine) and aminoglycosides (11%) [3, 4]. Of the antibacterials used in horses in Spain, 2% were classified AMEG category B (restrict use), 13% category C (use with caution) and 85% category D (prudent use). The Spanish dashboard present pharmaceutical forms as injectables (43%), dermatological (37%) and oral (20%), without the possibility to separate between for example oral paste and oral powder.

Indicators for antibacterial use vary across studies. The study from the United Kingdom applied DDDvet per animal per year [7], whereas others, including our study, used the proportion of treatment occasions, records or amount of active substance [3–6, 8]. DDDvet, developed by EMA, is a standardised metric facilitating comparisons across animal species, countries and antibacterial classes. One DDDvet represents the assumed average maintenance dose for one day for a medication used for its main indication in a specified animal species. The use of DDDvet is strongly recommended because it enables harmonisation of reporting and allows meaningful benchmarking, accounting for differences in potency and dosing regimens. However, we did not report DDDvet for three main reasons: (1) EMA has not yet developed DDDvet values for horses; (2) the DDDvet used in the study from the United Kingdom were based on the average daily dosage in its own dataset, which may not reflect Norwegian practices, and they applied the same DDDvet for all antibacterials in a class and did not differentiate by pharmaceutical form; and (3) estimating the DDDvet per animal per year requires accurate population data, which remains uncertain in Norway due to probable underreporting in the National Horse Registry regarding births, imports and deregistration of deceased horses [31, 32]. The National Horse Registry contained 85 993 live horses in April 2024 and 78 528 live horses under the age of 35 years per March 2025 [32, 33]. The uncertainty related to these population data in combination with the incomplete antibacterial use data is also why use is not presented in mg active substance per kg animal biomass in the current study. A potential difference between years for this indicator could have been caused by variation in data quality as well as actual differences in use. Further use of the indicator mg/kg animal biomass to compare use across countries would have introduced another layer of insecurity as the method for calculating completeness might differ between countries and studies. This is also not recommended in the EMA report on antibacterial sales and use for animals in 2024 [34].

When prescribing medications, veterinarians are required to assign at least a general organ-related diagnostic code for the indication, while providing a more specific disease code is voluntary [35]. The treatment guidelines for antibacterial use in horses gives guidance on choice of antibacterial for specific diagnoses or conditions [12]. Hence, general organ-related diagnostic codes are insufficient for assessing adherence to these guidelines in detail. In VetReg, the proportion of records with only a general organ-related code was high, ranging from 77% in 2022 to 72% in 2024, with higher proportions for pharmacies (> 90% in all years) than for veterinarians (39–51%). This limits the ability to evaluate guideline adherence at an overall level. Nevertheless, specific cases can still highlight prudent or non-prudent use. For example, specific diagnosis codes for endometritis and metritis were too rarely used in our data to generally evaluate prudence of use for these diseases. The nine reported cases of endometritis were treated systemically (i.e. oral paste or injections), while the treatment guidelines suggest local treatment if bacterial infection is detected [12]. Because the general treatment of gynecological and obstetrical conditions was in accordance with other treatments using antibacterials, reported treatments of these conditions alone do not indicate that stud farms in Norway are a particular risk for AMR transfer compared to other horse facilities with similar flow of horses.

It was not feasible to assess prudent use for AMEG category A antibacterials because no records were assigned a disease specific code. There is a diagnosis (*Rhodococcus equi* infection) where rifampicin is a suggested treatment in the therapeutic guidelines. For AMEG category B antibacterials, nine records were assigned colic/abomasum displacement as disease code. These are conditions generally not treated with antibacterial agents, although, they can be symptoms of conditions that should be treated with antibacterials. Another limitation when evaluating prudent use based on VetReg data is that only the first diagnosis assigned by the veterinarian in the veterinary practice management software for a consultation is transferred to VetReg via the animal health data platform owned by Animalia (the Norwegian Meat and Poultry Research Centre) [36]. Any additional diagnoses are not captured. Veterinarians may alternatively report their information directly to VetReg through the Norwegian Food Safety Authority’s web portal. When using the web portal, the veterinarians can only assign one diagnosis per reporting, but in this situation the veterinarians are actively selecting the diagnosis they are reporting. In our data, 4% of the records reported by veterinarians were reported through the Norwegian Food Safety Authority’s web portal. However, it is likely that most consultations have either only one diagnosis or when multiple diagnoses are assigned the main diagnosis is stated first and will be the one registered in VetReg.

In Norway there is unfortunately a lack of extensive data related to AMR among clinical isolates from equines. Although screening of the normal horse population is regularly performed in the national monitoring-program NORM-VET [19]. In those screenings, MDR bacteria are generally uncommon with 0–1% carriage of ESBL/pAmpC *E. coli* and only one case of MRSA in 2017. The low carriage is likely associated with that horses in Norway have not been treated with third or fourth generation cephalosporins. Use of fluoroquinolones, macrolides, tetracyclines, aminoglycosides, and penicillins (predominantly beta-lactamase sensitive penicillins) was low during the study period, with 4.4% of the amount of active substance used in 2022, 4.5% in 2023 and 5.0% in 2024. These were almost exclusively (> 90%) given as injections, which is one of the pharmaceutical forms considered to have the lowest impact on AMR selection [14]. There is however a potential for import of horses with AMR-bacteria from other European countries. This is because import of horses and periods abroad for Norwegian horses is possible [37]. In contrast, import of food-producing animals to Norway is almost non-existing, except for the import of poultry [38]. In the latest screening on horses in Norway, carriage of methicillin-sensitive *S. aureus* (MSSA) in addition to MRSA, was also included (Börjesson et al. Manuscript in preparation). No MRSA was detected but around 20% of the MSSA isolates were resistance against benzylpenicillins, with other antibacterials only occasionally showing reduced susceptibility [19]. As the current study found that sulphonamide and trimethoprim are the classes primarily used for horses in Norway, it is worrisome to note an increasing trend of reduced susceptibility to these antibiotics for faecal *E. coli* isolated from the general horse population within NORM-VET [19]. In 2024, 19% and 18% of the *E. coli* isolates showed reduced susceptibility to sulphonamide and trimethoprim (2009: < 10%), respectively. Although among *S. aureus* in the general horse population reduced susceptibility to trimethoprim appears uncommon (Börjesson et.al. Manuscript in preparation). One could suspect that resistance among clinical isolates might be higher for both bacteria, but confirmed data on clinical isolates from horses in Norway is generally lacking. The reduced susceptibility to sulphonamide and trimethoprim can be compared to that in Germany, which has comparatively lower use, where 30–40% of pathogenic *E. coli* isolates showed reduced susceptibility between 2020 and 2023 [39]. Another reason for the choice of active substances in Norway, in addition to low prevalence of AMR, might be the limited number of antibacterial veterinary medicinal products for systemic use marketed in Norway for horses: seven products per 26.02.2026 [40]. These products belong to only four classes of active substances: Natural penicillins, sulphanomides, trimethoprim and aminoglycosides. According to EU Regulation 2019/6, Articles 112 and 113, marketed products for the relevant species and indication should be first choice for treatments [23].

VetReg is technically prepared to capture all data required for reporting antibacterial use in horses from 2026 onwards, in accordance with the EU regulation 2019/6 [23]. The overall completeness of use data increased from 75% in 2022 to 85% in 2024, while pharmacy data completeness increased from 76% in 2022 to 96% in 2024. This is higher overall completeness than what is reported in previous quality assessment of VetReg data from 2016, 2023 and 2024 [12, 25, 41]. These previous completeness assessments have included records for antibacterial veterinary medicinal products used in horses (2016-data), cattle, pigs and poultry, while in this current study we included data on antibacterial veterinary medicinal products predominantly used in horses. The high completeness observed for horse data is largely driven by oral paste, which has consistently shown a high completeness in previous quality assessments. In contrast, completeness for injections in our study was substantially lower than previously reported. Our completeness evaluation for injections was solely based on a gentamicin product only used in horses, which may explain this discrepancy. Gentamicin is part of the treatment guidelines for some acute diseases such as septicaemia in foals and sepsis in synovial structures [12]. It is therefore likely that horse veterinarians buy the medicine as preparedness for acute conditions that may or may not occur in their practice.

Only a limited proportion of all antibacterial treatment records missed information on animal species (2022 and 2023: 0.1%; 2024 < 0.1%). This could stem from either the veterinarian’s prescribing or the pharmacy’s entry of their dispensing. Missing information on animal species means that those records cannot be used in analysing animal species specific use and reporting to EMA. Information on the number of horses treated was missing for approximately half of the records, with higher proportion of missing in records from veterinarians than from pharmacies. This is an important limitation and is most likely a consistency issue where information is not transferred correctly between the various software included. It is likely that the initial veterinary record in the PMS contained information on number of horses as horses mainly are treated individually and records are kept per individual horse. For horse data it is reasonable to assume, as we did in this study, that one horse was treated in the case of missing information.

Unit used to report the amount used was not pre-defined for any medicinal product or pharmaceutical form. Consequently, various units have been reported for the same medicinal product and form. Correct unit reporting is essential for ensuring accurate estimation of amounts used. No unit errors were observed for injections, but errors were found in 1% of the records for oral paste. These proportions were lower than those reported for VetReg antibacterial data in general [25, 41]. This issue could be reduced by implementing pre-defined units in the veterinary practice management software.

Another accuracy concern was reporting of presumably incorrect amounts, which could involve both higher and lower amounts than actual use. Excluding such records during data cleaning would underestimate the calculated use compared to actual use. Therefore, we adopted a conservative approach using high cut-off values for data exclusion. The cut-off values were based on dosing information in the summary of product characteristics, and when horse-specific dosing information was missing we used cattle information as a proxy. This is a limitation as dosing may differ between horses and cattle, and horse-specific dosing information might be available in the literature. However, this was relevant for a low amount of antibacterials in each year, ranging between 0.5 and 1.3 kg active substance per year. In Denmark, the VetStat system addresses this issue by providing all reporting veterinarians with a feedback report on their use data and requiring correction of identified errors [42], a process that likely improves data quality. Implementing similar quality control measures in Norway would require regulatory changes and new technical solutions.

## Conclusion

Antibacterial use pattern in horses in Norway was assessed to be similar to patterns described from Germany and the United Kingdom, but somewhat different from the most recent data published from Estonia and Spain. The use was relatively stable in the period when evaluated as kg active substance. Further development in the DDDvet methodology, to also cover horses, and better population data is needed to fully take into account various dosing regimens between medicine formulations and between active substances. The use in general and for gynecological and obstetrical conditions specifically do not select directly for the highly unwanted AMR such as against third and fourth generation cephalosporins and/or carbapenems. The results from this study warrant further studies related to use and resistance to sulphonamide and trimethoprim. Prudent use could be evaluated to some extent, but the reporting using general organ-related diagnosis limited this evaluation. Therefore, clinical records must be studied to fully evaluate prudent use and to investigate which diseases should be attempted prevented to reduce the need of antibacterials in horses. The quality of VetReg data of antibacterial use in horses were evaluated to be in line with or better than for those species for which data are already reported to EMA.

## Supplementary Information

Supplementary Material 1.

Supplementary Material 2: Supplementary Figure 1. Diagnostic groups assigned for treatment with AMEG B antibacterials in horses in Norway, 2022-2024.

## Data Availability

The data used in this study are not publicly available. The data were available for the Norwegian Veterinary Institute via an agreement with the Norwegian Food Safety Authority and the Norwegian Institute of Public Health. The data can also be requested by others from the same providers.
