# Supplementary material for: Antibacterial use in Norwegian horses: a nationwide registry-based cross-sectional study
Source: BMC Vet Res. 2026 Apr 16;22:282. doi: 10.1186/s12917-026-05474-z (PMC13191911; doi:10.1186/s12917-026-05474-z)
Supplement: Supplementary file 2 — Supplementary Material 2: Supplementary Figure 1. Diagnostic groups assigned for treatment with AMEG B antibacterials in horses in Norway, 2022-2024. [file 12917_2026_5474_MOESM2_ESM.pdf]

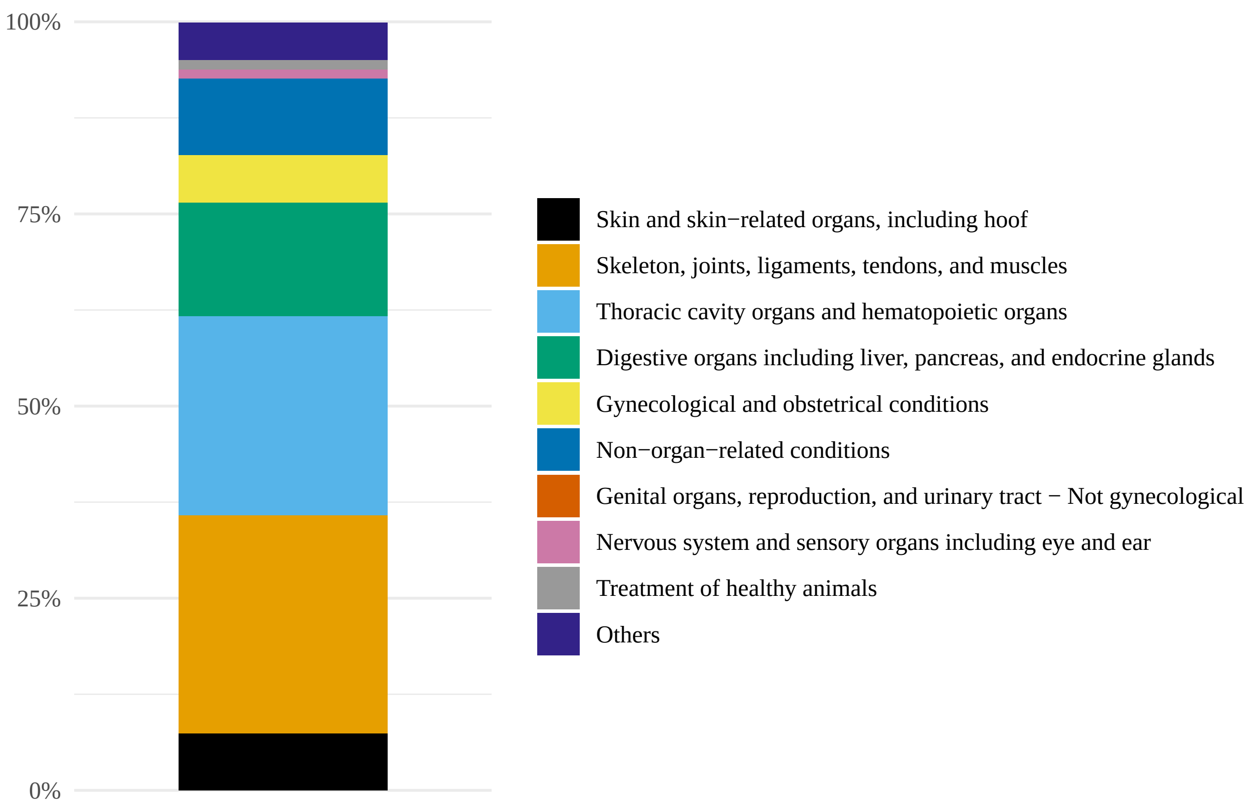


Diagnostic groups assigned for treatment with AMEG Bantibacterials in horses in Norway, 2022-2024 Percentage of records assigned the diagnostic group. Data comes from the Veterinary Prescription Registry. AMEG (Antimicrobial Advice Ad HocExpert Group). Skin and skin-related organs, including hoof (7.4%) Skeleton, joints, ligaments, tendons, and muscles (28.4%) Thoracic cavity organs and hematopoietic organs (25.9%) Digestiveorgans including liver, pancreas, and endocrine glands (14.8%) Gynecological and obstetrical conditions (6.2%) Non-organ-related conditions (9.9%) Genital organs, reproduction, and urinarytract - Not gynecological (0.0%) Nervous system and sensory organs including eye and ear (1.2%) Treatment of healthy animals (1.2%) Others (4.9%)
